# Comparing Retinal Structure in Patients with Achromatopsia and Blue Cone Monochromacy Using OCT

**DOI:** 10.1016/j.xops.2021.100047

**Published:** 2021-07-28

**Authors:** Emily J. Patterson, Christopher S. Langlo, Michalis Georgiou, Angelos Kalitzeos, Mark E. Pennesi, Jay Neitz, Alison J. Hardcastle, Maureen Neitz, Michel Michaelides, Joseph Carroll

**Affiliations:** 1 UCL Institute of Ophthalmology, University College London, London, United Kingdom; 2 Moorfields Eye Hospital, London, United Kingdom; 3 Ophthalmology and Visual Sciences, University of Louisville, Louisville, Kentucky; 4 Casey Eye Institute, Oregon Health & Science University, Portland, Oregon; 5 Ophthalmology, University of Washington, Seattle, Washington; 6 Ophthalmology & Visual Sciences, Medical College of Wisconsin, Milwaukee, Wisconsin; 7 Cell Biology, Neurobiology & Anatomy, Medical College of Wisconsin, Milwaukee, Wisconsin

**Keywords:** Achromatopsia, Blue cone monochromacy, Cone, Ellipsoid zone, Fovea, Foveal hypoplasia, Hyper-reflective band, Imaging, OCT, Photoreceptor, SD-OCT, X-linked cone dysfunction, ACHM, achromatopsia, BCM, blue cone monochromacy, ELM, external limiting membrane, ERG, electroretinography, EZ, ellipsoid zone, LCR, locus control region, LRP, longitudinal reflectivity profile, NPV, negative predictive value, PPV, positive predictive value, SD-OCT, spectral-domain OCT

## Abstract

**Purpose:**

To compare foveal hypoplasia and the appearance of the ellipsoid zone (EZ) at the fovea in patients with genetically confirmed achromatopsia (ACHM) and blue cone monochromacy (BCM).

**Design:**

Retrospective, multicenter observational study.

**Participants:**

Molecularly confirmed patients with ACHM (n = 89) and BCM (n = 33).

**Methods:**

We analyzed high-resolution spectral-domain OCT (SD-OCT) images of the macula from patients with BCM. Three observers independently graded SD-OCT images for foveal hypoplasia (i.e., retention of ≥1 inner retinal layers at the fovea), and 4 observers judged the integrity of the EZ at the fovea, based on an established grading scheme. These measures were compared with previously published data from the patients with ACHM.

**Main Outcome Measures:**

Presence of foveal hypoplasia and EZ grade.

**Results:**

Foveal hypoplasia was significantly more prevalent in ACHM than in BCM (*P <* 0.001). In addition, we observed a significant difference in the distribution of EZ grades between ACHM and BCM, with grade II EZ being by far the most common phenotype in BCM (61% of patients). In contrast, patients with ACHM had a relatively equal prevalence of EZ grades I, II, and IV. Grade IV EZ was 2.6 times more prevalent in ACHM compared with BCM, whereas grade V EZ (macular atrophy) was present in 3% of both the ACHM and BCM cohorts.

**Conclusions:**

The higher incidence of foveal hypoplasia in ACHM than BCM supports a role for cone activity in foveal development. Although there are differences in EZ grades between these conditions, the degree of overlap suggests EZ grade is not sufficient for definitive diagnosis, in contrast to previous reports. Analysis of additional OCT features in similar cohorts may reveal differences with greater diagnostic value. Finally, the extent to which foveal hypoplasia or EZ grade is prognostic for therapeutic potential in either group remains to be seen, but motivates further study.

Achromatopsia (ACHM) and blue cone monochromacy (BCM) are 2 congenital cone dysfunction syndromes that are of great interest due to the emergence of novel therapeutic approaches leading to clinical trials. Although patients with ACHM typically lack function of all 3 cone types, patients with BCM retain function of their short-wavelength–sensitive cones (which comprise only 7%–10% of the normal total cone population). Although ACHM is autosomal recessive and BCM is X-linked, the inheritance pattern is not always clearly discernible, especially in smaller families with few affected individuals. Moreover, clinical symptoms are similar between the 2 pathologies, and inconsistent nomenclature throughout the literature poses a further challenge to their differentiation.^1^, ^2^, ^3^, ^4^ As a result, diagnosis is not straightforward, particularly in clinics that do not have access to, or funds for, genetic testing or other specialized assessments. Accounting for the estimated prevalence of the known underlying genetic causes of ACHM (40%–50% *CNGB3*; 20%–30% *CNGA3*; < 2% *GNAT2*,^5^ *PDE6C*, and *PDE6H*),^6^^,^^7^ it is estimated that the genetic cause of at least 15% of ACHM cases remains unknown (although some of these cases may represent missed intronic variants or even misdiagnosed BCM);^8^ thus, there is a need to develop methods to better differentiate these conditions clinically.

Literature examining clinical differences in these populations is sparse,^9^, ^10^, ^11^ especially in molecularly confirmed patients. Some differences in visual function have been found between ACHM and BCM, but with limited discriminative abilities. Differences between these groups have been found in eye movements using electro-oculography,^12^ as well as in cone responses using electroretinography (ERG),^4^^,^^11^ although ERG presentation in BCM and both *GNAT2-* and *PDE6C*-related ACHM can be similar due to preservation of short-wavelength sensitivity.^13^^,^^14^ Moreover, the procedures are not feasible for all patients, especially children, and photopic ERG stimuli can be particularly uncomfortable for some patients, due to the photoaversion that is characteristic of both conditions.

Color vision testing can offer a less vexatious alternative, with differences between ACHM and BCM being evident on the Sloan ACHM test,^15^ although with limited reliability, as well as the Berson test.^10^^,^^16^^,^^17^ However, the accuracy of any functional test is dependent on patient concentration and cooperation. Even for patients who perform reliably, detection of any subtle differences in visual performance requires specialized expertize and equipment, specific lighting conditions, and calibration of stimuli, making such methods impracticable in most clinics. However, methods to assess cone structure that are widely available, less dependent on patient performance, and readily interpreted, may offer an alternative approach for discriminating BCM from ACHM.

Spectral-domain OCT (SD-OCT) is used widely in clinical settings and enables visualization of the retinal layers as distinct reflective bands. The second hyperreflective outer retinal band has been shown to correspond to photoreceptor integrity, and the reflective signal has been hypothesized to originate from the mitochondria-rich ellipsoid zone (EZ) or the junction between the inner and outer segment of photoreceptors. For simplicity, we refer to the second band as the “EZ.” Discontinuities in the EZ have been observed at the fovea in patients with BCM, suggesting disruption of photoreceptor structure.^11^^,^^18^, ^19^, ^20^ Likewise, there is variable disruption of the EZ at the fovea in patients with ACHM (ranging from normal-appearing to complete absence). Although this variability does not correlate with visual function,^21^ it does broadly correlate with remnant foveal cone density, as assessed using adaptive optics imaging.^22^ Comparison between the 2 pathologies using longitudinal reflectivity profile (LRP) analysis of time-domain OCT images showed reduced total foveal thickness in BCM compared with ACHM,^11^ although subsequent SD-OCT studies have reported retinal thinning in both BCM and ACHM.^18^^,^^23^ In addition, Barthelmes et al^11^ reported an absence of the EZ in ACHM and an absence of the external limiting membrane (ELM) in BCM, suggesting this is an absolute biomarker for distinguishing the 2 conditions. Of note, the patients used in that study were not genotyped, but instead were classified using best-corrected visual acuity, ERG, and color-plate testing.

We use SD-OCT to assess foveal hypoplasia and the appearance of the EZ at the fovea in patients with genetically confirmed BCM, and compare with previously reported data from patients with genetically confirmed ACHM.

## Methods

### Patients

Images from 33 male patients with genetically confirmed BCM were used for analysis. The genotype and clinical phenotype for each patient are shown in Table 1. Thirteen patients had a deletion of the locus control region (LCR), and 20 patients had the Cys203Arg substitution affecting the only opsin gene or at least the first 2 genes in the *OPN1LW/OPN1MW* array. The LCR deletions preclude expression of all *OPN1LW/OPN1MW* genes, whereas genes with the Cys203Arg mutant encode a nonfunctional opsin that is toxic to the cones that express it. The ACHM data for 89 patients were drawn from 2 previously published studies: 38 patients with *CNGA3*-related ACHM (21 male; 17 female) from Georgiou et al^24^ and 51 with *CNGB3*-related ACHM (30 male; 21 female) from Langlo et al.^22^ This study followed the tenets of the Declaration of Helsinki and was approved by local Institutional Review Boards (MCW: PRO17439 & PRO30741; UCL/Moorfields reference: 67979). Informed consent was obtained from all patients after the nature and possible consequences of the study were explained.

**Table 1:** Summary of the Genotype and Clinical Phenotype of Subjects with Blue Cone Monochromacy

Family	Subject	Age (yrs)	Disease-Causing Variant	Eye	OCT Grade	Foveal Hypoplasia
F1	JC_0078	27	LCR deletion	OS	III	No
F2	MM_0223	13	LCR deletion	OS	II	Yes
F3	JC_0611	34	LCR deletion	OD	III	Yes
F4	JC_0613	14	LCR deletion	OD	II	No
F5: IV-1	JC_0909†	7	LCR deletion	OS	II	Yes
F5: III-4	JC_0911†	41	LCR deletion	OD	II	No
F5: II-8	JC_0912†	58	LCR deletion	OS	IV	No
F6	KS_10992	25	LCR deletion	OD	II	No
F7	JC_11033	53	LCR deletion	OS	III	No
F8	JC_11230	8	LCR deletion	OS	II	Yes
F9: IV-3	JC_11237†	6	LCR deletion	OD	II	Yes
F9: II-1	JC_11239†	75	LCR deletion	OS	III	No
F9: III-8	JC_11266†	35	LCR deletion	OS	II	No
F10	MM_0151	54	M_C203R_	OD	V	No
F11	MM_0177	10	M_C203R_	OD	I	No
F12	JC_0183∗	24	M_C203R_	OD	II	No
F12	JC_0184∗	21	M_C203R_	OS	II	No
F13	MM_0187	21	M_C203R_	OD	I	Yes
F14	MM_0235	16	M_C203R_	OD	II	No
F15	JC_11532∗	49	M_C203R_	OS	II	No
F15	JC_11585∗	54	M_C203R_	OS	IV	No
F16: IV-1	JC_10066†	24	L_C203R_-L_C203R_	OS	II	No
F16: IV-3	JC_10067†	13	L_C203R_-L_C203R_	OD	II	No
F16: III-7	MP_10100†	35	L_C203R_-L_C203R_	OS	II	No
F17: IV-7	MP_10097†	43	L_C203R_-M_C203R_	OS	II	Yes
F17: V-2	MP_10116†	10	L_C203R_-M_C203R_‡	OS	I	Yes
F18	MM_0186	11	M_C203R_-M_C203R_	OD	II	No
F19	JC_0440∗	18	M_C203R_-M_C203R_	OD	II	Yes
F19	JC_0441∗	18	M_C203R_-M_C203R_	OS	II	No
F20	JC_10557∗	16	M_C203R_-M_C203R_	OS	IV	No
F20	JC_10558∗	16	M_C203R_-M_C203R_	OD	II	Yes
F21	JC_10561	50	M_C203R_-M_C203R_	OS	IV	Yes
F22	JC_11919	20	M_C203R_-M_C203R_	OD	I	No

C203R = Cys203Arg; LCR = locus control region; OD = right eye; OS = left eye.

For simplicity, only the first 2 genes within the *OPN1LW/OPN1MW* array are reported.

∗ The following are brothers: JC_0183 and JC_0184; JC_11532 and JC_11585; JC_0440 and JC_0441; JC_10557 and JC_10558.

† Pedigrees shown in Supplemental Figure 1 (available at www.ophthalmologyscience.org).

‡ Genotype inferred from MP_10097.

### SD-OCT Imaging

High-resolution SD-OCT images of the macula were acquired using the Bioptigen Envisu R2200 (MCW) or C2300 (UCL/Moorfields) SD-OCT systems (Leica Microsystems). High-density horizontal line scans (750 or 1000 A-scans/B-scan, 100–150 repeated B scans) were acquired through the foveal center. Line scans were registered and averaged to reduce speckle noise in the image, as previously described.^25^ Images from both eyes for each patient were reviewed by a single rater (E.J.P.), and the eye with better image quality was then selected for further analysis. For the patients with ACHM, SD-OCT images from the right eye of patients included in 2 previously reported studies were used for analysis.^22^^,^^24^

For the patients with BCM, foveal hypoplasia was assessed in a binary fashion (i.e., presence or absence) independently by 3 raters (E.J.P., C.S.L., M.G.), with the consensus grade being used for all images. For the patients with ACHM, their previously reported foveal hypoplasia status was used in our analysis. For the patients with BCM, the EZ integrity at the fovea was assessed by 4 raters (E.J.P., C.S.L., M.G., J.C.). We used Sundaram et al’s^21^ 5 categories for grading: (I) continuous EZ, (II) EZ disruption, (III) EZ absence, (IV) presence of a hyporeflective zone, or (V) outer retinal atrophy (including loss of retinal pigment epithelium). Any assessment that did not reach a consensus across raters was reviewed and discussed (by E.J.P. and J.C.) for a final determination. For the patients with ACHM, their previously reported EZ grade was used in our analysis. Statistical analysis was performed using GraphPad Prism (version 9.0.0, GraphPad Software), R (The R Foundation), and SAS (version 9.4, SAS Institute, Inc). A Shapiro–Wilk test was used to test for normality. Because the data were found to have a non-normal distribution, nonparametric tests were used to test for statistical significance.

## Results

Foveal hypoplasia judgements were identical between eyes for all BCM patients. The EZ grading was identical between eyes for all BCM patients except JC_11033, whose right eye was graded as grade V and left eye as grade III by a single rater (E.J.P.), demonstrating high interocular symmetry in BCM. The eye with better image quality was used for further analysis. Foveal hypoplasia judgments were also identical between eyes for all ACHM patients. Four of 51 ACHM patients had interocular differences in EZ grade, again demonstrating high interocular symmetry.

### Foveal Hypoplasia

**Figure 1:**
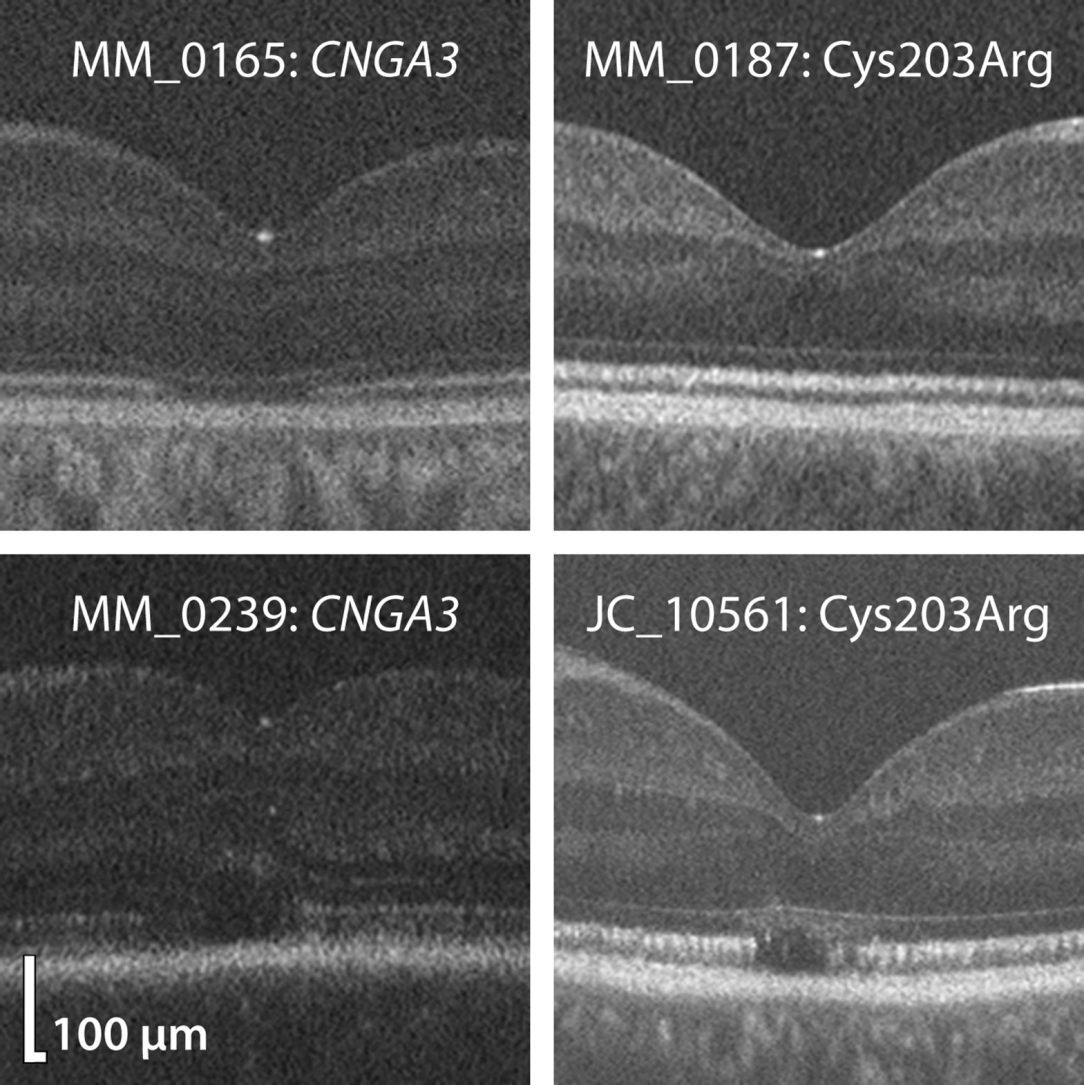
Examples of foveal hypoplasia in achromatopsia (ACHM) and blue cone monochromacy (BCM). Shown are processed Bioptigen spectral-domain OCT (SD-OCT) images of 2 patients with *CNGA3*-related ACHM and 2 patients with Cys203Arg-related BCM. Subjective assessment reveals that foveal hypoplasia is more severe in ACHM than BCM, because there is greater retention of inner retinal layers. Images in this figure were rotated to negate tilt for aesthetic purposes.

Sixty-two of the total 89 ACHM patients (70%) had foveal hypoplasia, compared with 11 of 33 BCM patients (33%). Examples of foveal hypoplasia in ACHM and BCM are shown in Figure 1. A Fisher exact test revealed that foveal hypoplasia was significantly more prevalent in ACHM than BCM (*P <* 0.001). Within each condition, we found no association between the underlying genotype and the prevalence of hypoplasia (ACHM: *CNGA3* vs. *CNGB3*, *P =* 0.64; BCM: LCR deletions vs. Cys203Arg, *P =* 0.71).

Given that the majority of ACHM patients had foveal hypoplasia and the majority of BCM patients did not, it was of interest to determine the predictive value of the presence of hypoplasia. The sensitivity of foveal hypoplasia as a diagnostic sign for differentiating between ACHM and BCM was 70% (95% CI, 59–78) and the specificity was 67% (95% CI, 50–80), with a positive predictive value (PPV) of 85% (95% CI, 75–91) and negative predictive value (NPV) of 45% (95% CI, 32–59).

### EZ Integrity

**Figure 2:**
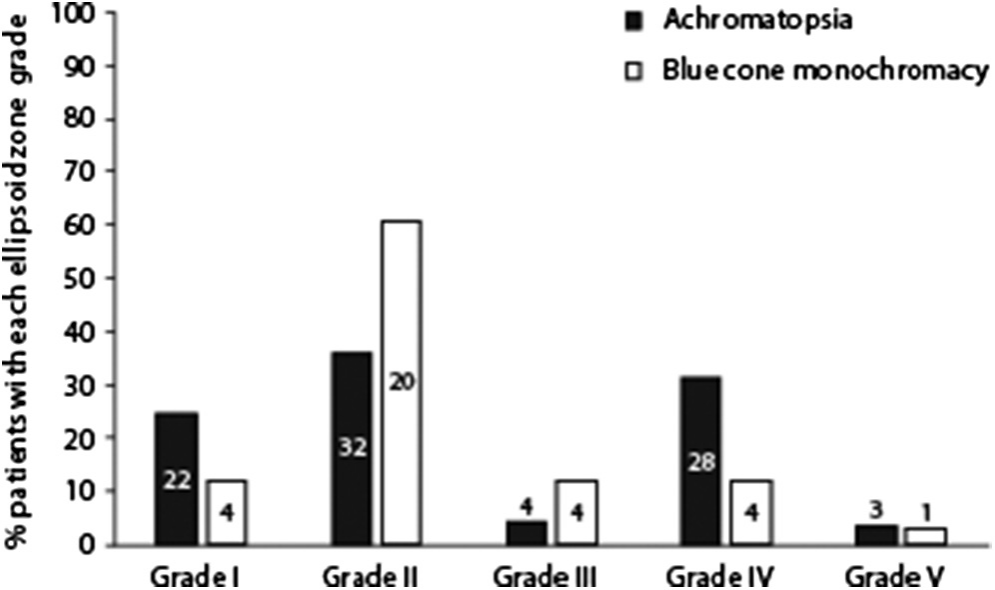
Percentage of each ellipsoid zone (EZ) grade in achromatopsia (ACHM) and blue cone monochromacy (BCM). The frequency of each grade is shown within or above each bar. We observed a significant difference in the distribution of grades between ACHM and BCM, with a grade II EZ being the most common phenotype in BCM. Patients with ACHM were more than twice as likely to have a grade IV EZ than BCM, suggesting that functional short-wavelength–sensitive cones in BCM may help to prevent development of a hyporeflective zone at the fovea.

A breakdown of the relative prevalence of the different EZ grades within BCM and ACHM is shown in Figure 2. Of note is the large proportion of BCM patients with grade II EZ (61%) compared with ACHM (36%), as well as the higher prevalence of grade I and IV in ACHM (25% and 31%, respectively) than BCM (12% and 12%), and of grade III in BCM (12%) than ACHM (4%). Grade V accounted for 3% of retinas for both ACHM and BCM. A Fisher exact test revealed a significant difference in the distribution of grades between pathologies (*P =* 0.02), with a Cramér’s V yielding a moderate effect size of 0.30.

Because of the low prevalence of EZ grades III and V, patients with these grades were excluded from the following analysis. The distribution of EZ grades between pathologies remained significantly different (*P =* 0.01, Pearson’s chi-square test), with a Cramér’s V yielding an effect size of 0.28. Grades I and IV were significantly more prevalent in ACHM than BCM (*P <* 0.004, Fisher exact test). The sensitivity of grades I and IV as a diagnostic sign of ACHM was 61% (95% CI, 50–72) and the specificity was 71% (95% CI, 51–87), with a PPV of 86% (95% CI, 75–94) and NPV of 39% (95% CI, 25–54).

Multivariable exact logistic regression showed that both hypoplasia (*P =* 0.004) and EZ grade (with 3 levels, *P =* 0.026) had significant predictive value when controlling for the other factor. The area under the curve in the multivariate model was 0.669 for hypoplasia (95% CI, 0.566–0.772), 0.667 for EZ grade (95% CI, 0.564–0.771), and 0.743 with both factors combined (95% CI, 0.642–0.844), which represented a significantly better predictive value than either factor alone (*P <* 0.0001). Examination of the classification table allows evaluation of sensitivity and specificity when using a decision rule based on a given cut-point probability of ACHM (Table 2).

**Table 2:** Classification Table from Multivariate Logistic Regression

Hypoplasia	EZ Grade	n	Predicted Probability of ACHM	Sensitivity	Specificity	PPV	NPV	Sensitivity + Specificity
No	I, II, or IV	23	0.4372	1.0000	0.0000	0.7455	--	1.0000
No	I or IV	11	0.7442	0.8780	0.4643	0.8276	0.5652	1.3423
No	IV	9	0.7510	0.7683	0.5357	0.8289	0.4412	1.3040
Yes	I, II, or IV	29	0.7567	0.6951	0.6429	0.8507	0.4186	1.3380
Yes	I or IV	15	0.9209	0.4268	0.8929	0.9211	0.3472	1.3197
Yes	IV	23	0.9235	0.2683	0.9643	0.9565	0.3103	1.2326

EZ = ellipsoid zone.

Rows are ordered by predicted probability of achromatopsia (ACHM). Sensitivity, specificity, positive predictive value (PPV), and negative predictive value (NPV) apply to a decision rule base on a cut-point probability. For example, a cut-point at *P =* 0.7567 predicts that all patients with hypoplasia and any EZ grade have ACHM with sensitivity = 69.5%, specificity = 64.3%, PPV = 85.1%, and NPV = 41.9%. A cut-point at *P =* 0.7442 minimized classification error (which is statistically optimal, although it may not be clinically optimal).

### Examining Possible Sex Differences

All BCM patients were male, so it was important to establish that sex differences in the ACHM group were not contributing to any differences found between conditions. A Fisher exact test showed no statistically significant difference in the prevalence of foveal hypoplasia between male and female patients across the ACHM group (*P =* 0.17). In addition, there was no significant difference in age between ACHM and BCM groups (*P =* 0.46, Mann–Whitney test). Thus the differences in hypoplasia and grade distribution between ACHM and BCM appear to be due to differences in the underlying disease mechanism.

## Discussion

**Figure 3:**
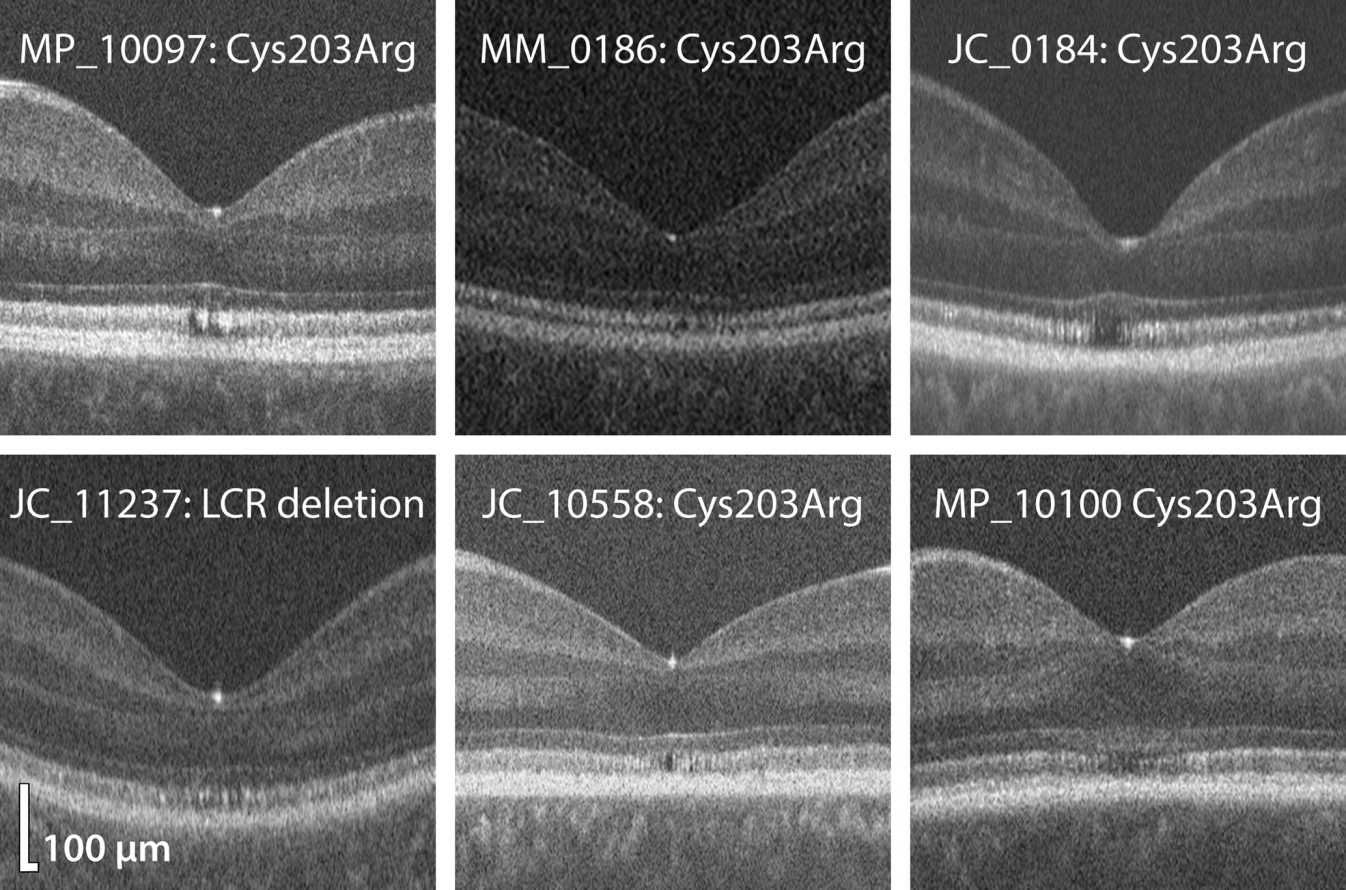
Examples of OCT images demonstrating the significant heterogeneity of grade II ellipsoid zone (EZ) in blue cone monochromacy (BCM). **MP_10097** and **JC_11237** are fairly typical examples of grade II, with both patients having disruption that extends the full height of the EZ, although MP_10097 has a focal disruption and JC_11237 shows broader mottling of the EZ. There was some debate as to whether **MM_0186** was grade I or II as, although there was a small focal disruption of the EZ just nasal of the foveal center, it did not extend the full height of the band. It was decided that any altered reflectivity constituted “EZ disruption.” **JC_10558** has a small pocket of hyporeflectivity, which may represent the short-wavelength–sensitive cone-free zone. There was contention between graders as to whether **JC_0184** was grade II or IV, as the region of hyporeflectivity is small, and it was debatable as to whether the ELM was bowing upward (which would indicate grade IV) or whether it had a normal contour (indicating grade II). Although BCM patients often lack the foveal bulge, it was decided that JC_0184 had a normal ELM contour. **MP_10100** had abnormal hyperreflectivity between the EZ and ELM, which gives the impression of a dipping ELM (perhaps indicating grade III), but it was decided that the ELM was intact, leaving the source of the abnormal hyperreflectivity unclear.

In this study, we compared patients with genetically confirmed BCM and ACHM to determine whether their SD-OCT images revealed distinguishable features that could aid differential diagnosis between the 2 patient populations. We found moderate differences in the distribution of EZ grades between ACHM and BCM, with ACHM patients being more likely than BCM to have grade I or IV EZ, and BCM patients being more likely than ACHM to have grade II or III EZ. In contrast to Barthelmes et al,^11^ who reported absence of the EZ (which they labeled P2) and presence of the ELM (which they labeled P3) in all ACHM patients, we observed several cases of EZ presence in ACHM and 3 cases of ELM absence (all grade V). The same study reported the opposite pattern for all BCM patients, a presence of the EZ (their P2) and absence of the ELM (their P3); however, we observed several cases of EZ absence and noted ELM presence in all but 1 BCM patient, who had macular atrophy (grade V). We believe that it is unlikely for all 6 of Barthelmes’ BCM patients to have lacked ELM while retaining EZ. Of the 4 bands they measured, the ELM (their P3) typically yields the smallest LRP peak; this, combined with the poorer lateral and axial resolution of time-domain OCT (compared with SD-OCT), as well as the inherent difficulty of obtaining sharp images in these populations, may have led to misindentification of retinal bands in some patients. In addition, they used the LRP at a single, precisely placed retinal location for grading the EZ, as opposed to the holistic EZ grading used in our study. Many BCM patients have a focal disruption of the EZ (Fig 3, JC_10558), which is hypothesized to represent the short-wavelength–sensitive cone-free zone,^18^ although this disruption does not always align axially with the foveal reflex (Fig 3, JC_0184), and therefore LRP analysis at the foveal center may miss a bona fide EZ disruption. More generally, dependence of LRP measurements on the precise placement of the LRP makes analysis susceptible to variation due to differences in signal, tilt in the OCT scan, or a lack of scanning frames at the exact foveal center. Furthermore, the steps required to overcome these issues often necessitate post-acquisition manipulation, which is not feasible in the clinic. Thus, although a categorical grading scheme has its own disadvantages, we think it provides a more accurate depiction of the EZ status of a given fovea than the isolated LRP approach.

We also found that patients with ACHM were significantly more likely to have foveal hypoplasia than patients with BCM. Barthelmes et al^11^ did not explicitly comment on hypoplasia; however, the broader internal limiting membrane peak (which they called “P4”) reported in ACHM than both normal and BCM suggests that their P4 may also have incorporated other inner retinal bands, such as the plexiform layers, thereby making it highly likely that hypoplasia was present in their ACHM population. The finding that foveal hypoplasia is more prevalent in ACHM than BCM has important implications for the mechanisms underlying human foveal development. In the immature eye, all retinal layers are still present at the fovea.^26^ Histologic and in vivo studies have shown a lateral shift of inner retinal layers away from the fovea in utero, which continues throughout the first few months after birth.^27^^,^^28^ Its failure to occur in most ACHM patients suggests that cone function helps to guide this process. Additionally, the finding that peripheral migration of inner retinal layers occurs in most BCM patients suggests that retained function of a single minority cone class may be sufficient to prevent severe hypoplasia. The fact that S-opsin expression precedes L/M opsin and rhodopsin expression, as well as foveal cone migration and Henle fiber elongation, lends support for this hypothesis.^29^^,^^30^

One issue raised in the process of conducting this study is the ambiguity in classifying OCT images. For example, the extent to which the EZ must be “disrupted” to warrant a grade II (as opposed to grade I) is arguable and, to some extent, arbitrary. Must the disruption extend the full height of the EZ band at the fovea (Fig 3, MP_10097 and JC_11237) or is it sufficient for it to simply have altered reflectivity (Fig 3, MM_0186)? Differentiating between grades II and IV can be particularly problematic. Literature using Sundaram et al’s^21^ grading scheme appears to classify a vitread bowing of the ELM (in combination with a hyporeflective zone) as grade IV, although this is not explicitly stated. One feature often observed in BCM is a small “pocket” of hyporeflectivity at or near the fovea (Fig 3, JC_10558); the threshold at which this pocket becomes a hyporeflective “zone” is not clearly defined. Moreover, many patients with BCM lack a foveal bulge,^20^ whereby the ELM inclines inward (i.e., upward in our images) at the foveal center. This feature (Fig 3, JC_0184), or lack thereof (Fig 3, MP_10100), may influence one’s interpretation of the term, “hyporeflective zone,” which is used to describe the foveal cavitation in grade IV. Therefore, this grading scheme may be less suitable for BCM than for ACHM in its current form, but could perhaps benefit from further clarification within each grading category. Foveal cavitation has been observed in a number of inherited retinal dystrophies,^31^^,^^32^ and is likely to be indicative of outer segment loss,^31^ rather than cone loss, as adaptive optics imaging has revealed remnant inner segments within these areas.^22^ Future work combining OCT with en face adaptive optics imaging may help to elucidate the cellular origin of abnormal patterns of reflectivity observed in OCT, particularly in the photoreceptor layers. Such clarity could facilitate the development of anatomically and clinically relevant grading schemes.

One notable limitation of the current study is that differences between pathologies may have been lost through binary classification of foveal hypoplasia. Although not assessed quantitatively, it was noted that there was a trend toward a greater number or thickness of preserved inner retinal layers at the fovea in ACHM than in BCM (Fig 1). Binary assessment not only ignores this potentially important difference but also increases uncertainty when categorizing images from BCM patients. Future work may benefit from quantifying the number or thickness of retained inner retinal layers, which could be facilitated by using directional OCT. The reflectivity of the Henle fiber layer changes depending on the pupil entry position, which could help to disambiguate hypoplasia judgments. Furthermore, given recent advances in deep learning techniques and their successful application to ocular images, it is also possible that by using training data consisting of SD-OCT images classified simply by genotype, a convolutional neural network may be able to distinguish between the pathologies.

Accurate diagnosis is critical, not only for the welfare of the individual patient but also for estimations of disease prevalence. There has been renewed interest in congenital cone disorders because of recent advances in gene therapy efforts to restore cone function. However, motivation to target a given disease will be influenced by its prevalence. The prevalence of each pathology has been somewhat “lost in translation” throughout the literature, no doubt exacerbated by ambiguous descriptions and use of terms,^1^, ^2^, ^3^^,^^33^ as well as a misunderstanding of the genetic origin in earlier work. Blue cone monochromacy has variably been referred to as “incomplete” or “atypical” ACHM, although both terms have also been used to describe different conditions. Estimates for “total color blindness” (i.e., ACHM and BCM combined) range from 1/20 000 to 1/100 000 of the total population,^33^^,^^34^ with the majority consisting of autosomal recessive ACHM.^1^ Blue cone monochromacy is generally considered to affect approximately 1/100 000 individuals,^35^ although early estimates quote as few as 1/100 million people,^2^ and even 1/100 million percent.^1^ Misdiagnosis of BCM for ACHM could potentially contribute to an underestimation of BCM, making it a less favorable target for gene therapy efforts. Therefore, it is crucial to ensure accurate diagnosis and to continually update estimates of prevalence based on emerging research.

In conclusion, despite our finding that the distribution of EZ grades is significantly different between diseases and that foveal hypoplasia is more prevalent in ACHM than BCM, these population differences likely cannot be used to definitively diagnose an individual patient, in contrast to previous reports.^11^ However, OCT findings could be used to guide diagnosis or decisions concerning genetic testing, as *OPN1LW*/*OPN1MW* sequencing is not widespread. Moreover, as our understanding of how OCT disruptions relate to the underlying cone structure improves, accurate classification/grading of images will be of great importance in interpreting progressive changes or responses to therapeutic intervention.
