# Supplementary figures and images for: Comparing Retinal Structure in Patients with Achromatopsia and Blue Cone Monochromacy Using OCT
Source: Ophthalmol Sci. 2021 Jul 28;1(3):100047. doi: 10.1016/j.xops.2021.100047 (PMC9521040; doi:10.1016/j.xops.2021.100047)

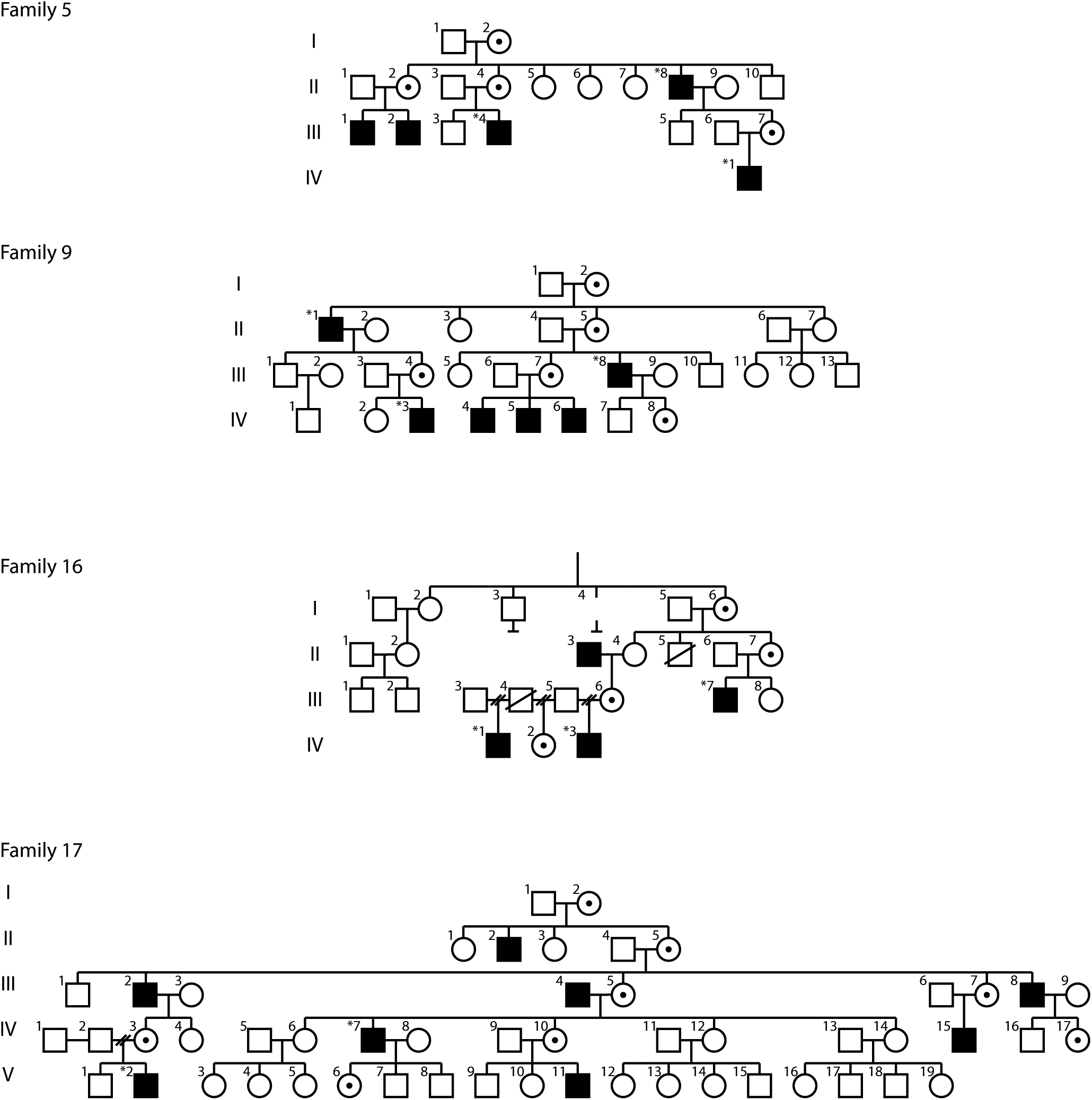

Supplement: Supplemental Figure 1 [file figs1.jpg]
